# Biochemical Traits in the Flower Lifetime of a Mexican Mistletoe Parasitizing Mesquite Biomass

**DOI:** 10.3389/fpls.2018.01031

**Published:** 2018-07-17

**Authors:** Elizabeth Quintana-Rodríguez, Alan Gamaliel Ramírez-Rodríguez, Enrique Ramírez-Chávez, Jorge Molina-Torres, Xicotencatl Camacho-Coronel, José Esparza-Claudio, Martin Heil, Domancar Orona-Tamayo

**Affiliations:** ^1^Departamento de Ingeniería Genética, Centro de Investigación y de Estudios Avanzados del Instituto Politécnico Nacional (CINVESTAV-IPN), Guanajuato, Mexico; ^2^Departamento de Soluciones Tecnológicas, Centro de Innovación Aplicada en Tecnologías Competitivas (CIATEC), Guanajuato, Mexico; ^3^Departamento de Bioquímica y Biotecnología, Centro de Investigación y de Estudios Avanzados del Instituto Politécnico Nacional (CINVESTAV-IPN), Guanajuato, Mexico

**Keywords:** floral phenology, volatile organic compounds, nectar chemistry, floral cell wall invertase, carotenoids

## Abstract

*Psittacanthus calyculatus* is a hemiparasitic plant that infects a wide range of trees. Mainly the biology reproduction of this mistletoe lies in bright colored flower development. Furthermore, it uses the nectar secretion as the only reward to engage different flower visitors. We investigated the physiological mechanisms of the flower phenology per hour and per day to analyze the spatial-temporal patterns of the nectar secretion, Cell Wall Invertase Activity (key enzyme in the quality of nectar), nectar chemistry, volatile organic compounds (VOCs) emission, synthesis of carotenoids and frequency of floral visitors. Flowers lasted 4 days, total nectar was loaded just before the anthesis and the secretion was maintained over day 1 and 2, decreased on day 3, and stopped on day 4. The diurnal nectar secretion dynamic per hour on day 1 and 2 showed similar patterns with high production on the morning and a decrease in the afternoon, the secretion declined on day 3 and ceased on day 4. On the other hand, CWIN activity per day was less before the anthesis and increased on day 1 and 2, this enzymatic activity decreased on the old flower phenology. Moreover, diurnal CWIN activities showed different patterns in the morning, noon, and lastly in the afternoon. Nectar chemistry varied significantly throughout of the flower lifetime, sucrose decreased along the flower phenology increasing glucose and fructose. Amino acids showed the prevalence of proline and oxo-proline, both increased on the day 1 and diminished in subsequent old flower stages. The spatial VOCs emission showed the presence of 11 compounds being β-ocimene the main volatile; its release increased on day 1 and remained constant in the flower lifetime. Lutein, lycopene, and β-carotene were concentrated in old stages of the flowers. In field, the most frequent flower visitors were the hummingbirds that usually foraging in all phenologic flower stage and their foraging events decreased with the phenological flower lifetimes. The results showed that these traits presented by *P. calyculatus* flowers are able to engage and manipulate the behavior of flower visitors and contribute to the reproduction of the parasitic plant.

## Introduction

The attraction of pollinators by flowers is based on different traits such as: floral nectar (FN) secretion, volatile organic compounds (VOCs) emission, and the production of color compounds in the flowers, these features are called pollination syndromes (Knudsen and Tollsten, 1993; Faegri and Van der Pijl, 2013). These pollination syndromes, as well as floral longevity and phenology are involved in the pollinator attraction behavior and floral specialization (Fenster et al., 2004; Guerra et al., 2014). Highly rewarding plants are common in a community with high diversity and they produce unique signals to ensure pollination (Schiestl and Johnson, 2013; Dar et al., 2017). These signals encourage pollinators to establish recurrent visits on flowers of these species, leading into fitness advantages in terms of increased receipt and export of intraspecific pollen to pollinate different flowers that results in the reproduction of plants (Wright and Schiestl, 2009; Rosas-Guerrero et al., 2014).

Flowering plants used different strategies to produce showy colored flowers with high secretion of FN (Zimmerman, 1988; Lucas-Barbosa, 2016). The latter is used as a unique reward to “manipulate” the pollinator behavior during and immediately following plant visits, affecting positively the pollen transfer and therefore plant reproduction (Aukema, 2003; Janovský et al., 2017). These plants offer high amounts of FN secretion (Ramírez and Ornelas, 2010) rich in sucrose and amino acids, which is biochemical adapted to the pollinator attraction (Heil, 2011). Furthermore, high quality nectar synthesis requires a complex enzymatic machinery. The first Arabidopsis flowers’ gene that encodes an apoplastic Cell Wall Invertase (CWIN) has been reported, this gene is compulsory to upload sucrose from the phloem and catalyze the hydrolysis of sucrose into glucose and fructose in nectar solution (Ruhlmann et al., 2010). Also, CWIN is important in the partition of sucrose in the extrafloral nectar of *Acacia cornigera* (Orona-Tamayo et al., 2013) and *Ricinus communis* (Millán-Cañongo et al., 2014). The FN quality increases when it contains different amino acid concentrations such as proline, an energetic amino acid common in FNs, this amino acid is involved in the flying maintenance of insects and hummingbirds (Carter et al., 2006; Nepi et al., 2012). A classic example of interactions between the flowering plants that include these traits to attract pollinators is the well-known interaction between hummingbirds with mistletoe plants. Mistletoes comprise an aerial parasitic plants composed of around of 1,500–1,600 species worldwide (Nickrent et al., 2010). They are present in a variety of forms and are exclusively found in the tropical native species of South and Central America, Africa, Australia, and New Zealand (Fadini et al., 2018). These plants usually present different traits to manipulate the pollinator behavior that include: extended flowering lifetime (Azpeitia and Lara, 2006), *production of high quality FN* (Rivera et al., 1996; Pérez-Crespo et al., 2016), *VOCs emission* (Bungert et al., 2002; Sipes et al., 2014) and the developments of bright colored flowers (Pérez-Crespo et al., 2016). Among mistletoes the Loranthaceae family is one of the largest and the most diverse (73 genera and ∼990 species) (Nickrent et al., 2010).

The genus *Psittacanthus* is one of the most spectacular parasitic plants (∼120 species) that distributed from Mexico to the northern of Argentina (Kuijt, 2009). These mistletoes are found in 25 Mexican states situated in the central and southern regions (Azpeitia and Lara, 2006). Some of mistletoe species are totally dependent on bird pollination for their reproduction as reported for *P. calyculatus* (Azpeitia and Lara, 2006), *P. shiedeanus* (Ramírez and Ornelas, 2010), *P. robustus* (Guerra et al., 2014) and *P. auriculatus* (Pérez-Crespo et al., 2016) all of them show the syndrome of ornithophilus species. *Psittacanthus calyculatus* (DC.) G. Don (Loranthaceae) is an American mistletoe commonly found from Mexico to Venezuela (Azpeitia and Lara, 2006). In the central region of Mexico, this mistletoe parasitizes mainly mesquite (*Prosopis laevigata*) an endemic tree of this region. This parasite has become one of the main menace to the existence and death of mesquites, because the tree does not resist living with the mistletoe, however, a kill mesquite is used as biomass to produce different co-products.

We used *Psittacanthus calyculatus* that parasitizing *Prosopis laevigata* biomass to evaluate the floral lifetime and used this phenology to evaluate the spatial-temporal patterns of the nectar secretion, CWIN activity in the nectar secretion, nectar chemistry, VOCs emission, synthesis of carotenoids and frequency of floral visitors to link the flower traits with a strategy of mistletoe reproduction.

## Materials and Methods

### Study Area and Plant Material

The experiments were performed in a population of *Prosopis laevigata* (mesquite) highly infected with *P. calyculatus* located in a suburban area near of Irapuato in the state of Guanajuato in Central Mexico (20°43′ N; 101°19′ O at 1,730 m a.s.l). The weather of the area is mainly mild and humid, but dry at the end of each year. The rainy season is present in summer, with a mean annual precipitation of 650 mm and temperature of 18°C. All plants and flowers used showed no visible signs of infection by phytopathogens or damage by herbivores. Nectar quantification, and collection of flowers were conducted from June to September of 2015. Material collected to analyze the CWIN activities, nectar chemistry (sugars and amino acid compositions), carotenoid content, and floral visitor’s quantification were performed in 2016.

### Floral Phenology

We built up a categorization in order to classify the phenology. For this experiment, floral buds were selected in 10 mistletoe plants. The floral longevity and petal color were recorded during 5 days after petal flower excision. A semi-open bud was classified as “day 0” (D0), and subsequently, flower opening widely was designed as day 1 (D1), early young stages were designed as day 2 (D2) and day 3 (D3), mature stages were recorded as day 4 (D4), however, on day 5 (D5) petals had fallen and this stage was not taken for experiments. Only active flowers with nectar secretions were selected to analyze on the subsequent experiments.

### Temporal Patterns in Floral Nectar Secretions

The experiments were based on Azpeitia and Lara (2006) with modifications. Prior to the FN quantification, close and semi-open buds including the leaves were placed inside mesh bags to avoid floral visitors and they only were removed as soon as the nectar was quantified. Nectar was extracted from buds and flowers were measured at intervals 07:00, 09:00, 11:00, 13:00, 15:00 and 17:00 h during five consecutive days without removing the flowers. In a separate experiment, FN was collected at 07:00 during five consecutive days. FN was removed using a micropipette of 20 μl of volume, and the concentration of soluble solids was quantified with a temperature-compensated hand refractometer (Atago Co., Japan) as described earlier (Heil, 2004; Orona-Tamayo et al., 2013; Millán-Cañongo et al., 2014). To recover and record the nectar volume on the refractometer the nectar was collected using 5 μl microcapillaries. Rate secretions were calculated separately from the different stages. In all cases, different flower stages were collected after last collection of FN and oven-dried at 60°C to relate the FN amounts (as soluble solids) to the dry mass of the secreting flower by hour.

### Determination of Sugars and Amino Acids From the Floral Nectar Secretion

Nectar was collected and pooled from 10 plants and stored in a 1.5 ml in water-ice, after the nectar collection this was immediately frozen and stored at −70°C until further analysis. For the analysis of free sugar and amino acids we followed the methods described by Pais et al. (1986) with some modifications. For sugars, 10 mg of nectar was lyophilized, resuspended with 1 ml ultra-pure water, and the solution was passed through a cationic exchange Dowex 50w-x8 column (Bio-Rad, Hercules, CA, United States). Column was washed four times with 1 ml water and the aqueous solution containing sugars were collected and evaporated to dryness in a rotator evaporator. The amino acids retained in resin were eluted with the addition of four times of 1 ml of 4 M NH_4_OH. The solution was collected and processed as described earlier.

Nectar sample compounds were processed by reaction with addition of 20 μl pyridine and 80 μl *N,O*-bis(trimethylsilyl)-trifluoroacetamide (BSTFA), the mixture was incubated for 30 min at 80°C. After this time, 1 μl of each sample were analyzed by a gas chromatography system (Agilent 7890A; Agilent Technologies, Santa Clara, CA, United States) coupled to a mass-selective detector (Agilent 5975C; Agilent Technologies, Santa Clara, CA, United States) with a capillary column (60 m × 250 μm × 0.25 μm coating; Agilent Technologies, Santa Clara, CA, United States). Helium was used as carrier gas with a flux of 1 ml/min and the following temperature program was used: initial temperature at 70°C for 5 min and ramped at 5°C/min until 310°C for 15 min. The initial temperature of the injector was of 250°C. Carbohydrate and amino acids standards were prepared using the same methodology. Sugar and amino acid were identified using the National Institute of the Standards and Technology version 2.0 (NIST).

### Cell Wall Invertase Activity on the Floral Nectar Secretion

The floral nectaries are found in the base of the calyx of the flower of *P. calyculatus* (Galetto et al., 1990), and it has been reported that in these structures occur the presence of the CWIN (EC 3.2.1.26) which is a key enzyme involved in the quality, production and responsible for the hexose-rich composition of the FN (Ruhlmann et al., 2010). CWIN activity from the floral nectaries of *P. calyculatus* was determined in two independent experiments, one resembling the conditions as in the experiment designed to determine the time course in FN secretion each 2 h, and the other one resembling of FN each 24-h. All experimental conditions were as mentioned above. Only flower calyx that contains the floral nectary tissues were collected and pooled from 10 randomly selected plants for each experiment and finally they were immediately frozen in dry ice. Other flower parts were discarded (Azpeitia and Lara, 2006). Enzymatic activity was quantified as described by Orona-Tamayo et al. (2013) and Millán-Cañongo et al. (2014) with some minor modifications. Ground tissue (25 mg) was mixed with 5 mg of polyvinylpyrrolidone (PVP) and then with 500 μl of ice-cold 50 mM HEPES-NaOH (pH 8.0, containing 5 mM MgCl_2_, 2 mM EDTA, 1 mM MnCl_2_ and 1 mM CaCl_2_). Samples were incubated on ice for 10 min and then centrifuged at 10,000 × *g* for 20 min at 4°C. The supernatant was discarded and the pellet containing the cell walls associated invertases was washed three times with 500 μl of extraction buffer by re-suspending and centrifugation as described above. Finally, pellets were washed with 500 μl of ice-cold 80 mM sodium citrate, pH 4.8 and the invertase activity was measured as described previously (Orona-Tamayo et al., 2013; Millán-Cañongo et al., 2014) with some modifications. In short, 300 μl of 80 mM sodium citrate (pH 4.8; room temperature) were added to the pellets and the mixture was incubated at 37°C. The tubes were then centrifuged at 10,000 × *g* for 1 min at room temperature and 20 μl of each sample was mixed with 180 μl of HK reaction solution [Glucose (HK) Assay Kit Product Code GAHK-20, Sigma-Aldrich]. After reaching the steady state, 100 μl of an aqueous 100 mM solution of sucrose was added to the samples and the absorption was immediately measured at 340 nm in a μQuant^®^ Microplate-reader continuously. Aliquots were taken every 20 min for 80 min and analyzed as before.

### VOCs Emitted From the Floral Phenology

Volatile collections were performed in the different phenological flower stages that were mentioned above. However, due to asynchronous development of the *P. calyculatus* flowers, bunches only presented one, two or three stages that precluded the analysis of VOCs *in situ*. We collected and pooled 10 flower per stage and were placed into a 50-ml Erlenmeyer flask with tap water (1 cm of deep) and immediately were enclosed with aluminum foil and Parafilm^®^, and the VOCs were adsorbed using a Solid Phase Micro-Extraction (SPME; 2 cm, carboxen/Polydimethylsiloxane/Carbowax; Supelco, Bellefonte, PA, United States). Fibers were exposed by a period of 6 h. After this time, fibers were desorbed for 30 s into the GC-MS, and the program temperatures for separation were as follows: 60°C through 80°C at 5°C/min; 210°C at 8°C/min maintained at 210°C for 5 min (Quintana-Rodriguez et al., 2015). VOCs were identified using the NIST library.

### Carotenoids Accumulation in Flower Phenology

Determination of carotenoids in petals from the floral phenology were determined according to the methods of Li and Beta (2012) with some modifications. Petals (10 flowers) were detached and calyx and anthers were discarded, these were frozen and grinding. Samples were protected from the light and lyophilized.

To determine the total floral carotenoid contents, all manipulations were performed under dim light to avoid the minimal photochemical degradation. Tissue (0.1 g of petals) were extracted using 1 ml of mixture of ethanol (100%) and 0.1% butylated hydroxytoluene (BHT), the mixture was transferred to a Recti-Vial (Pierce Co.), mixed and incubated by 10 min at 80°C. After this time, 20 μl of 20% KOH was added and mixture was incubated as before. The mixture was combined with 500 μl of hexane (100%) and 1 ml of water and samples were centrifuged at 12,000 × *g* for 5 min at 4°C. The supernatant was collected and the remained residue was re-extracted as before until the residue was colorless and supernatants were combined. Extraction solvent was combined with 50 μl of extract and the absorption was measured at 470 nm in a microplate format. Total carotenoids were calculated using the following equation and expressed as μg/g (Li and Beta, 2012).

$$\text{Total carotenoids }(\mu \text{ g}/\text{g})=(\text{Ab}*\text{V}*10^{6})/({\text{A}}^{1\%}*100\text{G})$$

Ab is the absorbance at 470 nm, V is the total volume of extract, A^1%^ is the extinction coefficient for a 1% mixture of carotenoids at 2500 and G is the sample in dry weight (g).

For carotenoid composition, the carotenoid extracts were separated and quantified on Ultra-High Performance Liquid Chromatography (UHPLC) (Agilent 1200 infinity LC systems, Agilent Technologies, Santa Clara, CA, United States) coupled with a photodiode array detector was used. Carotenoids were separated using a Zorbax Eclipse Plus C-18 column (2.1 mm× 5.0 mm, 1.8 μm) with a temperature maintained at 40°C. The separation was achieved by a main solvent composition as followed: 55% methanol/40% acetonitrile/5% dichloromethane/0.1% BHT. The solvents were filtered through 0.45 μm membrane. The system was run in isocratic mode with a flow rate was kept constant at 0.4 ml/min for a total run time of 10 min. The injection volume of each sample and standards was 1 μl and absorbance was measured at 450 nm. The identification of the carotenoids was based on the congruence of retention times with those of pure carotenoids standards.

### Floral Visitors Related to the Phenology Stages

The frequency of flower visitors on the floral phenology was based on methods reported by Azpeitia and Lara (2006) and Guerra et al. (2014) with modifications. Open-buds, young and old flowers from different bunches (10 plants) were pruned-off. Only closed-buds were placed into mesh bags and then labeled. As soon as the buds were open (D0), we begin our records, which actually started from 6:30 h to 17:00 h by five consecutive days. We performed 80 h of focal observations over different days (10 days) and they were performed by using binoculars (10-22x50; Nikon) at distances between 10 and 15 m from the plants.

### Data Analysis

Data were analyzed with Least Significant Difference (LSD) *post hoc* tests after analysis of variance (ANOVA) due they meet the assumptions of heterogeneity and homoscedasticity and normal distribution, and in the case of visitors frequency in flowers, we used a χ^2^-test to evaluate the similarity in the visitor frequency and were performed using the Statistical Package for the Social Sciences 17.0 (SPSS Inc., Chicago, IL, United States).

## Results

### Time-Course of Floral Phenology

**FIGURE 1:**
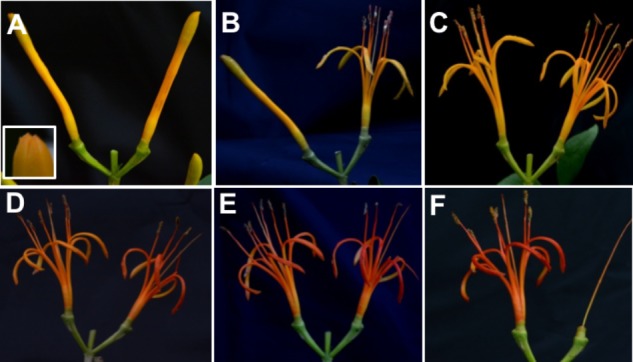
Time-course of flower stages. **(A)** Semi-opened bud on day 0 (D0) with an apical aperture on the tip. **(B)** The beginning of the flower anthesis on day 1 (D1); **(C)** day 2 (D2); **(D)** change of color on day 3 (D3); **(E)** day 4 (D4); **(F)** day 5 (D5).

*Psittacanthus calyculatus* flowers had a lifespan of 5 days, on the fifth day petals had fallen totally (**Figure 1**). In addition, petals showed a remarkable change of color through their phenologic stages. On D0, buds were partially open (semi-open bud) on the tip and presented a light yellow coloration, similarly color presented on D1, this color persisted on D2, while on D3 through D4 petals turned on a bright orange and on the D5 the petals fallen completely.

### Patterns of Floral Nectar Secretion

**FIGURE 2:**
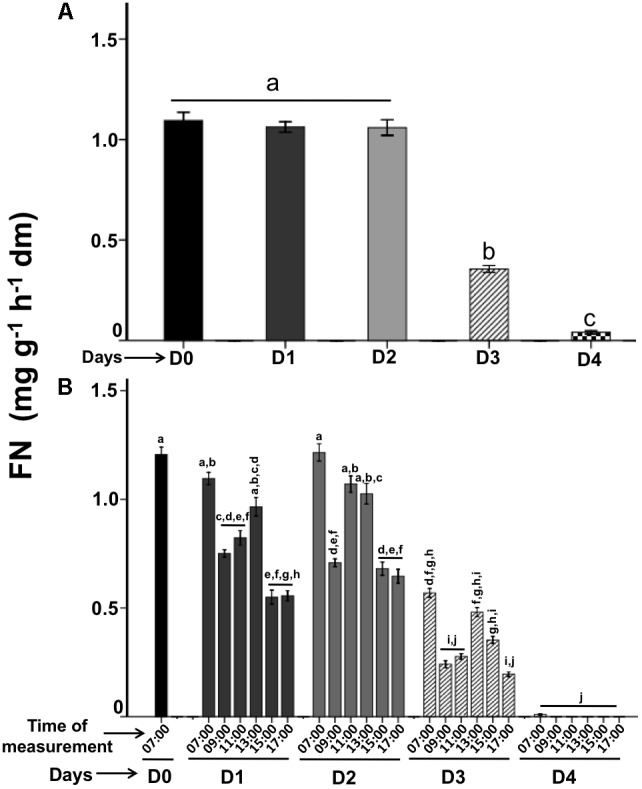
Dynamics of floral nectar secretion during the flower lifetime. **(A)** Floral nectar secretion was quantified each 24-h. **(B)** Diurnal floral nectar secretion measured each 2-h per day. Bars represent means ± SE (*N* = 66) of FN secretion (mg soluble solids per g flower dry mass and hour).

*Psittacanthus calyculatus* flowers produced high volume of nectar as a main pollinator’s reward. FN secretion per day collected at 24-h showed a high concentration of nectar in opened-buds (D0; 1.09 mg g^−1^ h^−1^ dm) collected at 07:00 am before the flower aperture (**Figure 2A**); this high nectar trend continued at the beginning of anthesis in the phenologic stage D1 (1.06 mg g^−1^ h^−1^ dm) and was maintained on the D2 (1.05 mg g^−1^ h^−1^ dm). However, the nectar secretion decreased on the D3 (0.35 mg g^−1^ h^−1^ dm) and dropped with the flower age on the D4 (0.04 mg g^−1^ h^−1^ dm) (*P* < 0.05) (**Figure 2A**).

The diurnal spatial patterns of nectar secretion collected each 2-h varied significantly (*P* < 0.05) in D1 (07:00–17:00) with concentrations of 1.1–0.55 mg g^−1^ h^−1^ dm, and increased again from 13:00 h (0.96 mg g^−1^ h^−1^ dm), and finally it decreased between 15:00 and 17:00 (**Figure 2B**). While on D2, the spatial nectar secretion was similar in amount and behavior to D1, FN was presented in high rate in the morning (07:00; 1,2 mg g^−1^ h^−1^ dm), decreased at 09:00 (0.70 mg g^−1^ h^−1^ dm), increased at the next times recorded (11:00–13:00; 1.0 mg g^−1^ h^−1^ dm) and showed a significant decrease at 15:00 (0.68 mg g^−1^ h^−1^ dm) and at 17:00 (0.64 mg g^−1^ h^−1^ dm) (*P* < 0.05). On D3 the nectar secretion showed a decrease in all times recorded: in the morning (07:00; 0.56 mg g^−1^ h^−1^ dm) presented a nectar reduction than the other days (*P* < 0.05), in the next points measured (09:00–11:00; 0.24–0.35 mg g^−1^ h^−1^ dm) it was more evident the nectar reduction. A dramatic nectar dropped was presented on D4 in all hours recorded, however in the morning (07:00; 0.001 mg g^−1^ h^−1^ dm) a significant nectar secretion rate (*P* < 0.05) was showed, but at followed hours the nectar was stopped completely (**Figure 2B**). In summary, the high nectar secretion rate was presented in the morning on the first 3 days.

### Patterns of Cell Wall Invertase Activity

Cell wall invertase is a β-fructofuranosidase that catalyzes the hydrolysis of sucrose into glucose and fructose (Roitsch and González, 2004). It has been suggested that this enzyme plays an important role in the nectar secretion (Ruhlmann et al., 2010; Orona-Tamayo et al., 2013; Millán-Cañongo et al., 2014). We used the above described FN patterns secreted per day and diurnal hours to investigate whether CWIN activity in floral nectaries are the main responsible for the FN secretion.

**FIGURE 3:**
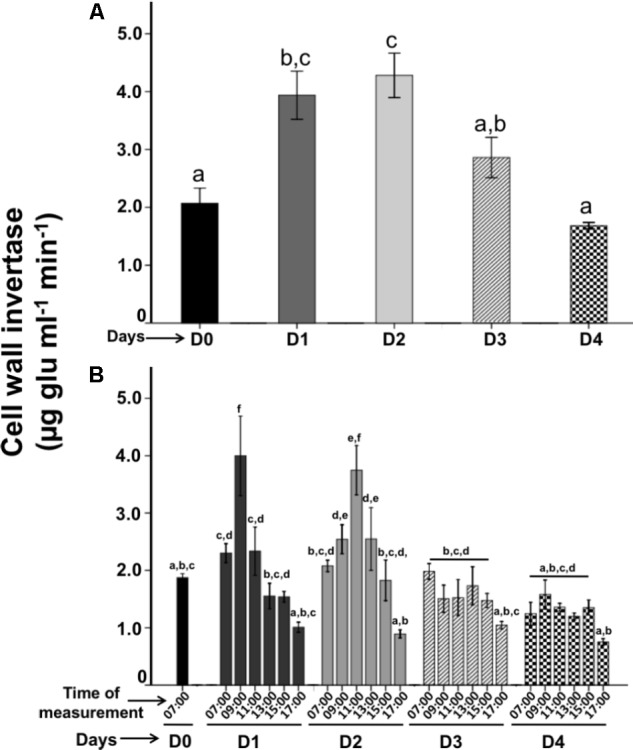
Time-course of Cell wall invertase (CWIN) activities during the active floral nectar secretion on the flower lifetime. **(A)** CWIN activities were quantified each 24-h during the active floral nectar secretion. **(B)** Diurnal CWIN activities were quantified during the floral nectar secretion measured each 2-h. Bars represent means ± SE (*N* = 3) of sucrose hydrolyzing activity (μg glucose released per min per ml).

Interestingly, on the phenological stages per day, we observed low enzymatic activity in opened-buds (D0; 2.07 μg glu ml^−1^ min^−1^; *P* < 0.05). However, when the flower anthesis begun, we found a CWIN high activity level between D1 and D2 (3.9 and 4.2 μg glu ml^−1^ min^−1^, respectively) (**Figure 3A**) that is related with the main peak of FN secretions (**Figure 2A**). On D3, CWIN displayed low activity (2.8 μg glu ml^−1^ min^−1^), on day D4 CWIN activity decreased (1.6 μg glu ml^−1^ min^−1^; *P* < 0.005). Therefore, the temporal patterns in the CWIN activities preceded the pattern in FN behavior by 2 h and these activities diminished due to FN display a reduction on the same phenological stages (**Figures 2A**, **3A**).

The CWIN activity (recorded each 2-h) varied significantly in diurnal hours. The D0 stage (07:00) showed a low enzymatic activity of 1.8 μg glu ml^−1^ min^−1^ (**Figure 3B**). However; CWIN activity increased on the next day D1 when the anthesis began; at 09:00 (4.0 μg glu ml^−1^ min^−1^) the highest activity on this day was recorded at 11:00 (3.7 μg glu ml^−1^ min^−1^), and it decreased gradually from 13:00 to 17:00 (2.5–0.9 μg glu ml^−1^ min^−1^). On D2, CWIN activity displayed similar activities to D1, in the morning (07:00) the activity was low (2.0 μg glu ml^−1^ min^−1^), then it increased at 09:00 (1.5 μg glu ml^−1^ min^−1^), and the highest activity was registered at 11:00 (3.7 μg glu ml^−1^ min^−1^), then it decreased gradually from 13:00 to 17:00 (2.5–0.9 μg glu ml^−1^ min^−1^). CWIN activities diminished on D3–D4 showing similar low enzymatic activities at 07:00 (1.9 and 1.2 μg glu ml^−1^ min^−1^, respectively), which gradually decreased from 09:00 to 15:00 (1.5 μg glu ml^−1^ min^−1^) to finally dropped to its lowest at 17:00 (1.0 and 0.75 μg glu ml^−1^ min^−1^; respectively). The CWIN activities per hour were more actives on D1-D2 than D3–D4, when these activities diminished, in parallel the nectar dropped, and the flower aged.

### Nectar Chemistry on Flower Lifetime

**FIGURE 4:**
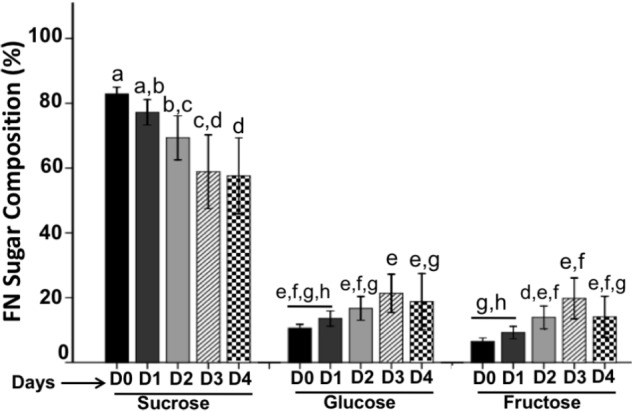
Sugar composition in floral nectar. FN is composed by sucrose as the main sugar followed by glucose and fructose. Bars represent means ± SE (*N* = 8) and reported as %.

Nectar sugar concentration varied throughout the flower lifetime. We found that sucrose was the dominant sugar followed by glucose and fructose both present in a similar concentration. Whereas sucrose concentration decreased gradually, glucose and fructose increased lightly as the flower age (**Figure 4**). Therefore, sucrose concentration was higher on D0 (83.0%; *P* < 0.05) than on the other days and glucose (10.5%) and fructose (6.5%) showed lower concentration. When the anthesis began on D1, sucrose decrease (77.2%) and glucose (13.6%) and fructose (9.22%) increase lightly. Similarly, on D2 the sucrose dropped (69.3%) and hexoses increased (glucose: 16.7%; fructose: 13.9%) and sugar reductions were more evident on D3 (sucrose: 58.8%) a showing increase of glucose (21.3%) and fructose (19.7%). Finally, the concentration of sucrose dropped on the D4 (57.6%), and hexoses decreased (glucose: 18.7%; fructose: 14.0%).

**FIGURE 5:**
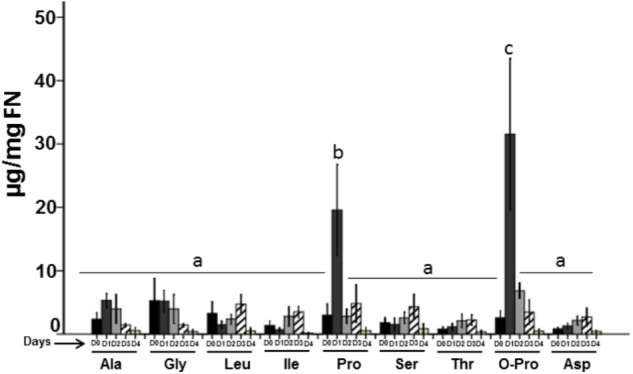
Total amino acids in floral nectar. The FN is composed by different amino acids, however, oxo-proline and proline were the most abundant. Bars represent means ± SE (*N* = 8) and are reported as μg/mg of nectar.

Nectar from the four flower stages studied (**Figure 5**) had a significant concentration of amino acids. We found alanine (Ala), glycine (Gly), leucine (Leu), isoleucine (Ile), proline (Pro), serine (Ser), threonine (Thr), oxo-proline (O-Pro), and aspartic acid (Asp). On D0 these amino acids showed similar concentration, however; on the next day (D1), Pro and O-Pro presented a higher concentration (0.19 and 0.31 μg/mg FN, respectively; *P* < 0.05), while the rest of amino acids did not increased their concentration (*P* < 0.05). On the D2, Pro (0.07 μg/mg FN) and O-Pro (0.03 μg/mg FN) showed a high reduction in their concentration. On the days D3–D4, Pro and O-Pro, as well as all amino acids, showed a more pronounced reduction.

### Volatile Organic Compounds Profiles From Flower Lifetime

In the floral VOCs profile, we identified 11 compounds from the *P. calyculatus* flower stages, and these compounds showed significant qualitative differences (**Table 1**). All of those compounds were present on the DO stage. However, only eight of those volatiles were present in the subsequent flower stages (see compounds in **Table 1**). On D2 six volatiles were present, while, on D3 only five compounds were collected and finally on D4 only two VOCs were identified. β-Ocimene was the main volatile presented through the flower lifetime; on D0, this volatile showed a value of 58.6% and increased on D1 (71.0%), on D2 this compound showed the highest concentration (94.2%) that dropped lightly (88.0%) on D3. The VOC 2,4-di-tert-butylphenol (DTBP) increased on the stage D1 (12.8%) and decreased on day D2 (2.1%) and disappeared on flower stage D3. Two compounds were emitted constitutively in all flower stages (β-ocimene and geranyl nitrile), while nonanol and β-farnesene were present only on stages D0 and D1 and disappeared in subsequent flower stages.

**Table 1:** VOCs emitted from the flower lifetime.

	Days				
Compound	D0	D1	D2	D3	D4
*Cis*-β-ocimene^∗^	58.6 ± 19.5b	71.0 ± 22.7b	94.2 ± 4.9a	87.9 ± 3.9b	94.8 ± 1.6a
Geranyl nitrile	9.0 ± 3.8b	5.2 ± 2.7b	2.2 ± 0.9a	6.9 ± 2.5a	2.6 ± 1.2a
1-hepten-4-ol	3.5 ± 1.8a	4.6 ± 1.9b	2.4 ± 1.6a	3.0 ± 1.1a	ND
*Cis*-hexenyl isovalerate	2.2 ± 0.8	ND	ND	ND	ND
*Cis-*3-hexenyl acetate	2.8 ± 1.2b	3.1 ± 2.0ab	2.3 ± 1.2a	1.4 ± 0.2b	ND
3-hydroxy-2,2,4-trimethyl pentyl ester of isobutanoic acid	5.9 ± 3.7	ND	ND	ND	ND
*Cis*-hexenyl butyrate	4.2 ± 2.4	ND	ND	ND	ND
Nonanal^∗^	2.1 ± 1.0a	2.8 ± 1.8a	ND	ND	ND
β-farnesene^∗^	2.9 ± 0.3a	2.7 ± 1.8b	ND	ND	ND
2,4-bis-(1,1-dimethylethyl)-6-methyl phenol	9.4 ± 1.3ab	12.8 ± 14.4ab	2.1 ± 1.7a	ND	ND
Neryl acetate	2.4 ± 1.3ab	1.3 ± 0.3ab	1.7 ± 2.0a	1.7 ± 0.6a	ND

*Compounds in the table are ordered according to their retention time. Total peak area percentage per gram of fresh weight of normalized five replicates are expressed in the table. Different letters represent statistically differences for each compound per day (*p* < 0.05 according *post hoc* Tukey’s HSD after ANOVA). ND, not detected. ^∗^Compounds identified with commercial standards.*

### Total Content of Carotenoid Composition on Flower Phenology

**FIGURE 6:**
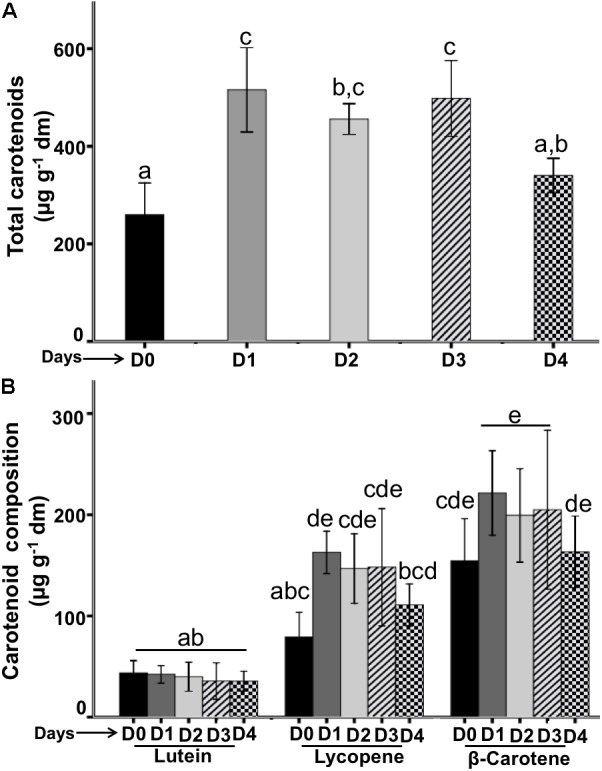
Quantification of carotenoids from flowers during the flower lifetime. **(A)** Total carotenoids contents were quantified each 24-h. **(B)** Composition of carotenoid types during the flower lifetime. Bars represent means ± SE (*N* = 6 for separately experiments) of carotenoids concentrations (μg per g flower dry mass).

The total carotenoids composition throughout the flower lifetime is shown in **Figure 6A**. On the stage D0 carotenoids content was lowest with a value of 259.7 μg/g dm, but this concentration increased and these values reached a maximum values on the next days D1–D3 (515.7–498.1 μg/g) and this concentration significantly dropped on D4 (340.1 μg/g).

Carotenoid composition on the flower phenology was composed by lutein, lycopene and β-carotene (**Figure 6B**). Lutein was the compound with the lowest concentration on all flower stages (D0–D4) that ranged from 43.7 to 35.6 μg/g. Lycopene was the second less concentrated compound in the flower lifetime, this compound on day D0 was present in a lowest concentration (79.1 μg/g) and increased on D1 (162.8 μg/g), its concentration was similar on the D2 and D3 (146.8–148.2 μg/g, respectively); and decreased on D4 (110.8 μg/g). β-carotene was the main compound presented in the flower lifetime, on D0 showed the lowest concentration (154.4 μg/g) that was increased on D1 (221.5 μg/g) and the concentration was similar on the subsequent days D2 and D3 (199.4–204.9 μg/g, respectively), but on D4 the concentration decreased significantly (110.4 μg/g).

### Visitors on the Flower Lifetime

**FIGURE 7:**
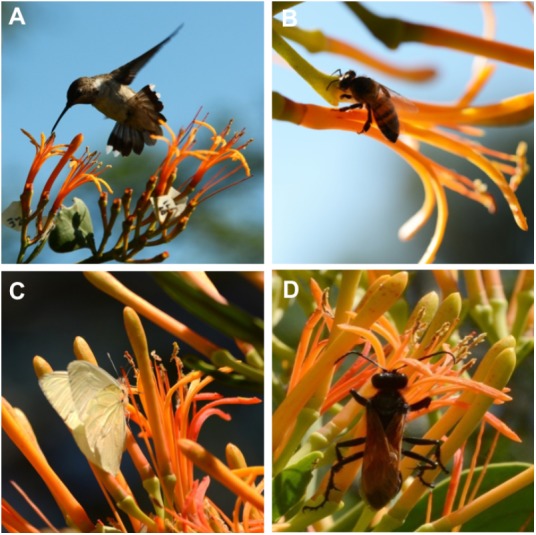
*P. calyculatus* visitors during different lifetime. **(A)** A *Cynanthus latirostris* hummingbird flying on flower on day 1. **(B)** *Apis mellifera* robbing floral nectar on day 2. **(C)** *Ascia* sp. butterfly accessing to the tube that contained floral nectar. **(D)** *Pepsis* sp. foraging behind of the mistletoe flower.

**FIGURE 8:**
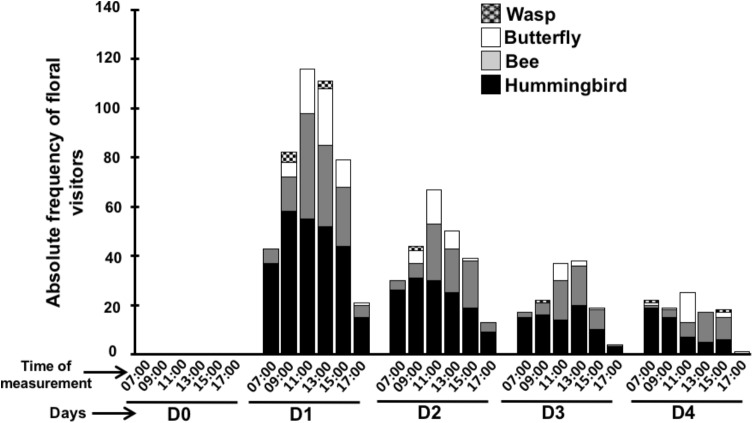
Visitors frequency in flower lifetime. Different consumer of floral nectar measured each 2-h during the flower lifetime.

To understand the relationship between flower phenology and visitors, we used the *in situ* phenology and recorded only four visitors: hummingbirds, bees, butterflies and wasps (**Figure 7**) that have been documented to be the regular visitors of *P. calyculatus* (Guerra et al., 2014; Pérez-Crespo et al., 2016). The main visitor in the different phenological flower stages were hummingbirds followed by bees, butterflies, and wasp (**Figure 8**). On day D0 we did not register visitors to search nectar, however on day D1, we recorded a total of 52 hummingbirds foraging events and their visits peaked actively between 09:00 and 15:00 h; the second visitors recorded were bees (25 foraging events) that actively visited flowers between 11:00 and 13:00 h, in both cases the visitors diminished at 17:00 h; butterflies were the third visitors (12 foraging events) with active hour from 11:00 to 13:00 and decreased at 17:00; wasps were occasional visitors (two foraging events) at 09:00, and between 13:00 and 15:00 h. On D2, we observed 28 foraging events by hummingbirds, beginning at 07:00, with its highest abundance peak at 09:00 and decreased on the following hours. Bees (15 foraging events) were more active between 11:00 and 15:00 h. While, butterflies (five foraging events) showed the highest abundance at 11:00 h and decreased at 13:00 h, and wasps visits (two foraging events) were occasional. On D3, all floral visitors diminish in its foraging events, we recorded that hummingbirds showed only 15 foraging events with a similar behavior on the hours recorded. However, we recorded an abundance on bee (nine visits), and butterflies only presented two foraging events, and an absence of wasps on all hour recorded. On D4, we observed a dramatically foraging events in all floral visitors, hummingbirds once again presented 10 foraging events showing its highest abundance peak at 07:00 h; bees visited this stage with only six visits with maximum abundance at 11:00 h, butterflies represent (four visits) being more abundant at 11:00 h, and finally we observed that the less abundant visitor were wasps (less of one foraging events). We note that all foraging events dropped in parallel with the phenological flower stages and flower visitors diminished their visits to the old flowers.

## Discussion

### Flower Lifetime of *P. calyculatus*

Flower phenology of *P. calyculatus* lasted 5 days of metabolic activity; in anthesis, the color changed from a light yellow, which persisted from D0 to D2. However, we observed a color change between D2 to D3 as a bright orange color, and on D4 petals changed totally to a bright-red; finally, on D5 petals had fallen. Similar results were found by Azpeitia and Lara (2006); however, they recorded 1 day more of floral longevity rather than our study. Other mistletoe flowers of the Loranthaceae family, showed different phenology stages such as *P. shiedeanus which* lasted 6 days (Ramírez and Ornelas, 2010), *Ligaria cuneifolia* lasted 4 days (Rivera et al., 1996), *P. robustus* lasted 3 days (Guerra et al., 2014) and *P. auriculatus* lasted 2 days (Pérez-Crespo et al., 2016). These differences can be due to the regional zones that presented environment changes that involve a high genetic divergence between mistletoe species.

### Floral Nectar Patterns on the Flower Lifetime

The dynamic of FN secretion from flowers in anthesis showed different amounts of nectar from semi-open bud (D0) through the old flower stages. Nectar secretion is loaded at the bud stage (D0), in this stage the bud is loaded with a high concentration of nectar; this active secretion continues for 48-h and decreased in old flower phenologic stage on D3 and become zero on D4. Azpeitia and Lara (2006) presented quantitative information on the FN secretion in *P. calyculatus*; they found that these flowers secreted high amount of nectar on the first 3 days and diminished in the last phenologic flower day, similar to our results. The high concentration of nectar in bud stage can be maintained due to a ready amount of nectar available for the consumption of floral visitors at the beginning of the anthesis. This could indicate an energy saving in the synthesis of nectar per day, which is highly expensive for the plant.

### Patterns on Cell-Wall Invertase Activity on the Flower Nectar Secretion

The spatial-temporal patterns of CWIN enzymatic activities resembles those of FN secretion; CWIN activity showed a low activity on D0 and become the highest peak on D1 and D2, this activity dropped on D3 and diminished close to zero on D4. Furthermore, the CWIN activities were similar across the diurnal secretion of nectar by 2-h. These CWIN activities are related to the replenishment of the nectar by day and hour. The FN of *P. calyculatus* contained high concentration of sucrose, but is would be expected the presence of a high hexose concentration; however, CWIN alone cannot be responsible for the differences in hexoses concentration and therefore other enzymes seem to play important roles in determining the FN sugar composition (Ruhlmann et al., 2010). Plants possess other types of invertases isoenzymes such as vacuolar invertase, neutral invertase and cell wall invertases. Alternatively, sucrose unloaded in the sink cells can be cleaved in the cytosol by neutral invertases or by vacuolar invertases, the hexoses resulting, by the activities of sucrose-cleaving enzymes can be used as substrates for different metabolic process (Roitsch and González, 2004). For example, vacuolar and neutral invertases activities seems to have a small influence on the hexose production and concentration regard in *Nicotiana attenuata* nectar (Tiedge and Lohaus, 2018). We do not discard these invertase activities and their influence in the metabolic modulation of sucrose in the nectary tissue of *P. calyculatus* flowers.

In fact, the sucrose came from the phloem, the CWIN enzyme can hydrolyze the sucrose to release glucose and fructose into the FN (Vassilyev, 2010); a general mechanism of nectar secretion could consist of the unloading of sucrose from the phloem via CWIN^1^ (Heil, 2015) and/or its synthesis in the floral nectary parenchyma with the aid of sucrose phosphate synthase and sucrose synthase, followed by its secretion into the extracellular space via SWEET9 and then its partial hydrolysis by an apoplastic invertase, which is eventually secreted into the liquid nectar (Heil, 2015). For example, in *Arabidopsis thaliana* mutant plants that lack CWIN gene resulted in a high sucrose concentration and a lower ratio of hexoses compared to the wild-type ecotype. In fact, in extrafloral nectaries of *Acacia cornigera* CWIN is active in previous hours of nectar secretion and diminishes its activity when nectar cease (Orona-Tamayo et al., 2013), similar CWIN activities occurred in extrafloral nectaries of *Ricinus communis* (Millán-Cañongo et al., 2014). Therefore, this enzyme is an important factor required for the nectar production.

### Nectar Chemistry on the Flower Lifetime

Chemical composition in nectar varies significantly throughout of the flower lifetime. There is a constant decrease in the sucrose concentration, however; glucose and fructose increased lightly as the flower phenology. Sugar composition of FN of *P. calyculatus* could be related with CWIN invertase activities that hydrolyze the sucrose releasing hexoses into the nectar. In bud stage (D0) we found a low CWIN activity and a high concentration of sucrose and lower concentrations of glucose and fructose. On the next days, the CWIN activities increased and the sucrose was hydrolyzed and then the hexoses were released into the nectar. This different sugar concentration found in FN in young flower stages could be due to the metabolic machinery rearrange; among the nectar constituent sugars are the most important, because they are the basis of the energy reward to different flower visitors (Baker and Baker, 1983) and this nectar sugar composition is related to the sucrose-cleaving enzymes. Sucrose is more attractive to pollinator birds and insects because these organisms prefer this sugar instead than monosaccharides, for example for hummingbirds, butterflies and other long-tongued bees usually prefer sucrose-rich FNs (Heil, 2011). In the case of FN of *P. calyculatus* flowers, the sucrose is the highest sugar present in all phenology stages this could be a pollination strategy for manipulate the attraction of flower visitors. In *Ligaria cuneifolia*, the sucrose diminished and hexoses increased with respect to flower lifetime (Galetto et al., 1990) similar than our results.

Nectar sugars are present between 100 and 1,000 times more than amino acids, and these can significantly affect the attractiveness of nectar (Heil, 2011, 2015). Certain amino acids are found frequently in different FNs (Baker and Baker, 1986) such as alanine, serine, proline, glycine, isoleucine, and threonine (Baker and Baker, 1973); all of these amino acids were found in *P. calyculatus* FN together with leucine, aspartic acid and oxo-proline. Proline and oxo-proline increased to a high concentration when the flower opened and decreased on the next days, this could be a strategy for pollinator attraction by the tasty nectar amino acids and sugars secreted by the flower. Proline is a normal constituent of many nectars and has also been identified at high levels in plant nectars and its function is due this amino acid can stimulate the insect’s salt cell concentration which results in an enhanced feeding behavior (Escalante-Pérez et al., 2012). Studies testing the feeding preference of forager honeybees for proline-, serine-, and alanine-enriched nectars, reported that proline-enriched nectar were preferred by these insects (Bertazzini et al., 2010; Noutsos et al., 2015). The presence of oxo-proline in nectar is a particular case, their presence at least is part of the different amino acid concentrations that are dissolved in the nectar solution; however, their function is involved in the glutathione metabolism, an important antioxidant found in plants, animals, and microorganisms (Gong et al., 2018), and their main function is to prevent the cellular oxidation caused by the reactive oxygen species such as free radicals and peroxides (Sabetta et al., 2017). Moreover, flight effort has been shown to increase oxidative stress levels in birds and insects (Janske et al., 2011) and probably when they seek nectar and consume it, their stress oxidative can be decreased mediated by the oxo-proline that promotes the glutathione synthesis a strong antioxidant in the muscles of the floral visitors. The presence of glucose, proline and oxo-proline in nectar represent a dual action, first proline is for rapid, short-term bursts of energy production and a large amount of glucose for extended flight (Carter et al., 2006) and oxo-proline an intermediate of glutathione that promotes antioxidant effects.

### VOCs Emitted on the Flower Lifetime

Flowering plants use a broad spectrum of signals to attract pollinators some of them are bright colors, and shapes to VOCs (Raguso, 2009). The VOCs profiles found in *P. calyculatus* revealed that the emission of these compounds tended to diminish through the time, being D0 where more compounds were found. Other studies showed that few compounds were found in the VOCs profile of plants pollinated mainly by hummingbirds (Cronk and Ojeda, 2008; Klahre et al., 2011). It has been reported that geranyl nitrile is emitted in petals, stamens, and calyxes of *Robinia pseudoacacia*, for this reason; we suggest that this compound remained present along the flower lifetime, since it can be emitted by distinct parts of the flower (Aronne et al., 2014). Interestingly, many VOCs were released on D0, several of these compounds are related with defensive functions and probably this is their main function in the bud and flowers of *P. calyculatus* (Schiestl, 2010). VOCs such as *cis-3*-hexenyl acetate, *cis*-3-hexenyl isovalerate, *cis*-3-hexenyl butyrate, and nonanal are compounds emitted commonly in response to bacterial or fungi diseases and they play a role as antimicrobial compounds (Pichersky and Gershenzon, 2002; Yi et al., 2009; Heil and Karban, 2010; Quintana-Rodriguez et al., 2015). β-Ocimene is a compound present in all floral phenology, it is a very common volatile released in flowers and has been reported to play multiple functions from the attraction of floral visitors to defensive functions (Farré-Armengol et al., 2017). Is important to note that the floral consumers such as insects, birds, and bats can transfer microflora among flowers, and other plant organs (Fridman et al., 2012), bacteria, fungi, and yeast in nectar may affect the nectar’s chemical composition, and thus reduced the pollination success (Vannette et al., 2013). Those volatiles emitted by *P. calyculatus* flower could be exerts an antimicrobial function rather than volatiles involved in the insect attraction or repellence. Future studies will allow determining if many of these compounds have antimicrobial activities.

### Carotenoids From the Flower Stages

Carotenoids are responsible for the yellow and red color of flowers and these compounds were changing in the flowering of the *P. calyculatus*. On the initial stages of the bud and anthesis, the flowers showed a yellow color on D0–D2, but on D3 the flowers presented a bright orange color and D4 they turned into a light red color. We can observe that on D2–D3 occur a transition change of yellow light to orange bright color and the flower become an orange intense as the amount of pigment increases; however, our carotenoids quantification did not fit with these flower color changes, only β-carotene the main pigment was constant in their concentration. Carotenoids are a large family of pigments, and are responsible for many of the brilliant red, orange, and yellow color in flowers (Delgado-Vargas et al., 2000). In addition, flowers can contain at least other important group of pigments such as anthocyanins, these are involved in the red color of the flowers (Miller et al., 2011), and we do not discard that these pigments are involved in the flower color transition of light to orange the flower of *P. calyculatus.* Azpeitia and Lara (2006) found similar flowering color pattern to our results, however, pigment petals did not were analyzed at the different floral stages. Color from floral parts constitutes the major visual attractants for pollinators (Ram and Mathur, 1984). When *P. calyculatus* flowers change in color after opening, the nature and biogenesis of floral carotenoids and their quantitative concentrations differ at different flower stages. The carotenoid contents that we found were lutein, lycopene, and β-carotene, lutein was the less concentrated at all flower stages; lycopene increased their concentration from D1 to D3 and diminished on D4. A similar behavior was observed for β-carotene, with a high concentration on D0, but a lower content of this pigment on D4. These patterns of carotenoids degradation can be due to the effect of pollinated flower (Ohmiya, 2013). Ram and Mathur (1984) evaluated the color changes subsequent to the anthesis and determined that the pollination was a key factor as a trigger for a rapid carotenoids and anthocyanin synthesis in the flower lifetime; for this reason, we can observed a carotenoid diminished in the lifetime of *P. calyculatus* flowers. Hummingbirds use their vision principally in finding yellow or bright red color flowers with copious quantities of FN (Miller et al., 2011). Schemske and Bradshaw (1999) found a weak correlation between hummingbird and bee visitors and the color of Monkey flowers (*Mimulus lewissi* and *M. cardinalis.*). In this research, hummingbirds did not exhibited preferences by flowers with similar concentrations of carotenoids or anthocyanins, and petal carotenoids significantly decrease the bee visitations, without effect on hummingbirds, concluding that the high concentration of these pigments function primarily to discourage bee visitation. However, in our field’s results we quantified a high percentage of bee visitation, these could be due to the effect of different VOCs that attract these insects. The ability of hummingbirds to quickly find rich nectar sources and to return to them suggests that hummingbirds are capable of exerting strong selection on the nectar rewards of flowers (Schemske and Bradshaw, 1999). These results are consistent with our findings due to the fact that hummingbirds do not have an innate preference for *P. calyculatus* yellow or red flowers and their attraction can be due to the high volumes of nectar in these flowers.

### Flower Visitors on the Flower Lifetime

Mistletoes of the Loranthaceae family are strongly associated with ornithophilia syndrome due to the flower morphology (tubular structures, colorful, robust corollas, and resistant sexual organs) (Faegri and Van der Pijl, 2013). We found that the *P. calyculatus* flowers received four types of floral visitors, hummingbirds, butterflies, bees and wasps. Hummingbirds were the main visitors, followed by bees, butterflies, and wasps. The foraging events of hummingbirds begin in the early hours of the morning and these are gradually reduced at midday; however, we found interestingly that as the visits of hummingbirds decreased, the visits of the insects increased, this could be to avoid conflicts of negative interactions between the nectar consumption. In addition, those foraging visits decreased in the different phenological flower stages and could be a direct effect of the nectar cease. Previous studies carried out on *P. calyculatus* found that its flowers are visited by four species of hummingbirds, observing a greater presence of visits also in the mornings and decreasing at the midday (Azpeitia and Lara, 2006) similar to our observations. The presence of hummingbirds as main pollinators in other mistletoe species such as *P. schiedeanus* (Ramírez and Ornelas, 2010), *P. robustus* (Guerra et al., 2014), and *P. auriculatus* (Pérez-Crespo et al., 2016) has been observed and therefore they have been cataloged as the main pollinators of these mistletoes, since they describe the hummingbird as an effective carrier of pollen (López de Buen and Ornelas, 1999). The hummingbirds’ head dimensions make them ideal for touching the anthers that contain the pollen, they carried the pollen on the head thus when visiting another flower, they can fertilize it. On the other hand, insects have a tiny size and they do not have effective contact with the anthers and pollination is not performed (Azpeitia and Lara, 2006). The presence of hummingbirds is related to the extended phenological flowering is seen as a pattern in mistletoes pollinated by these birds (Galetto et al., 1990; Rivera et al., 1996). It has been observed in different mistletoes that having high nectar production and flower longevity rates suggest a greater attraction of pollinators (Ramírez and Ornelas, 2010). The number of pollinator visits can be influenced by a large number of factors such as environmental conditions (temperature and humidity), attractions (visual and volatiles), and rewards (nectar and pollen) signals (Proctor et al., 1996). The plant ensures the attraction of pollinators through the anthesis of the flower by secreting large amounts of nectar rich in components such as sugars and amino acids that pollinator organisms seek to maintain their biological daily activities (Guerra et al., 2014). In that aspect, the hummingbird is possibly attracted by the sweet taste of the nectar derived from sucrose mainly, as well as by proline and oxo-proline.

In sum, the flowers of *P. calyculatus* contain different traits such as nectar production enriched with an excellent quality of biomolecules such as sucrose, glucose, and fructose, and energetic amino acids such as proline and oxo-proline as a reward for pollinators. The quality of sugar is due to the CWIN activity that biochemically is synchronized with the nectar secretion on the flower lifetime, the lower emission of VOCs was involved in the insect attraction, antimicrobial effects, or insect repellence, the showy color flower is composed by different carotenoids with functions of attraction and repellence. This is the first time that different traits are evaluated in this mistletoe flowers. These important suits can enforce pollination specificity and manipulate the behavior to improve the pollination in flowers of *P. calyculatus.*

## Author Contributions

EQ-R and DO-T designed the study, performed the experiments, and wrote the manuscript. AR-R and ER-C performed the experiments. XC-C performed the statistical tests. JM-T and JE-C provided equipment. MH provided intellectual suggestions.

## Conflict of Interest Statement

The authors declare that the research was conducted in the absence of any commercial or financial relationships that could be construed as a potential conflict of interest.

## Footnotes

1 http://www.youtube.com/watch?v=Nd8ryN_7BP8
